# Memory-making interventions for children and their families receiving pediatric palliative or bereavement care: A systematic review protocol

**DOI:** 10.12688/hrbopenres.13891.2

**Published:** 2024-07-17

**Authors:** Razieh Safarifard, Gemma Kiernan, Yvonne Corcoran, Eileen Courtney, John Mitchell, Terrah Akard, Veronica Lambert

**Affiliations:** 1 School of Nursing, Psychotherapy and Community Health, Faculty of Science and Health, Dublin City University, Dublin, Leinster, D09 V209, Ireland; 2 Head of Partnerships, Barretstown Children’s Charity, Ballymore Eustace, Kildare, W91 RDX6, Ireland; 3 School of Nursing, Vanderbilt University, Nashville, Tennessee, TN 37240, USA

**Keywords:** Memory-making, Intervention, Palliative care, Paediatric, Bereavement, Family support

## Abstract

**Background:**

In paediatric palliative and bereavement care, providing comprehensive support that extends beyond medical treatment to address the emotional and psychosocial needs of children and their families is essential. Memory-making interventions play a critical role in capturing cherished moments and fostering emotional resilience. However, widespread consensus on the foundation and scope of memory-making interventions for children and young people remains sparse. This review aims to identify, appraise, and synthesise the evidence on memory-making interventions for children and young people with life-limiting or life-threatening conditions and their family members receiving palliative or bereavement care.

**Methods:**

This systematic review will follow the Preferred Reporting Items for Systematic Reviews and Meta-Analysis (PRISMA). A systematic search will be undertaken from January 1, 1985, to February 27, 2024, across the following databases: PubMed, EMBASE, CINAHL (EBSCO), PsycINFO (EBSCO), Web of Science, the Cochrane Library, and Scopus. Studies across diverse research designs that examine children (0-19 years) with life-limiting or life-threatening conditions undergoing memory-making interventions with psychosocial or other outcomes will be included. Screening, data extraction, and quality appraisal will be performed by two independent reviewers, with a third reviewer resolving discrepancies. Joanna Briggs Institute guidelines for conducting mixed methods systematic reviews will be used to inform the data analysis and synthesis process.

**Conclusions:**

This review will provide critical insights into the existing evidence base on memory-making interventions in paediatric palliative and bereavement care, highlighting psychosocial and other impacts, implementation factors, and evidence quality. By identifying best practices and gaps in knowledge, this evidence review may inform future research and intervention design, or adaptation, and contribute to the enhancement of healthcare for children and young people with life-limiting and life-threatening conditions and their families as well as families in bereavement.

**Registration:**

This review was registered in PROSPERO, the International Prospective Register of Systematic Reviews (CRD42024521388; 18/03/2024).

## Introduction

The integration of paediatric palliative and bereavement care within the global health landscape is essential for providing comprehensive care to children facing life-limiting and life-threatening (LLLT) conditions, as well as their families. Despite the challenge of accurately estimating prevalence rates of children with LLLT conditions, the demand for palliative care services is significantly underestimated and is expected to rise ( Department of Health and Children (DoHC), 2009; Fraser *et al.*, 2021). Palliative care aimed at improving the quality of life for patients with serious illnesses and their families ( Hallenbeck & Hallenbeck, 2022). Children with life-limiting conditions have no reasonable hope of cure and are expected to die prematurely, while life-threatening conditions involve a high probability of premature death but may have feasible curative treatments ( Roland *et al*., 2022). Paediatric palliative and bereavement care services are crucial in circumstances where cure may not be an option, requiring care that extends beyond traditional medical treatments to address holistic care for children's physical, psychological, and spiritual needs, along with supporting families from diagnosis through end-of-life care or ongoing treatment ( Mitchell *et al.*, 2020; Together for Short Lives, 2018; World Health Organization, 2020). This aligns with the Sustainable Development Goal 3, which emphasises universal health and well-being, including palliative care as an essential service for achieving global health objectives ( United Nations, 2023).

Current estimates suggest around 21 million children globally require palliative care annually, with approximately 8 million needing specialist care ( Connor *et al*., 2017). In England, the prevalence of children with life-limiting conditions rose from 26.7 per 10,000 in 2001/02 to 66.4 per 10,000 in 2017/18, with the highest rates in the under 1-year age group ( Fraser *et al*., 2021). Similarly, in Queensland, Australia, the prevalence increased from 35.2 per 10,000 in 2011 to 43.2 in 2016, especially among children under one year and Aboriginal and Torres Strait Islander children ( Bowers *et al*., 2020). This growing demand highlights the importance of comprehensive palliative and bereavement care with the prevalence of children requiring these services expected to rise due to advancements in medical care and increasing recognition of paediatric palliative needs ( Roland *et al*., 2022).

Globally, the provision of bereavement support tailored to the needs of families of children receiving palliative care is recognised as a critical component of comprehensive care ( Applebaum *et al.*, 2023; Hudson *et al.*, 2018; Thornton *et al.*, 2021). This support, crucial for navigating the emotional, psychological, and practical challenges following the loss of a child, emphasises family-centred psychosocial interventions to enhance life quality and well-being ( Akard *et al.*, 2018; Benini *et al.*, 2022; Pedraza *et al.*, 2024). Family distress is a concern in paediatric palliative and bereavement care, where emotional burden can be overwhelming for parents ( Bronsema *et al*., 2022; Hasdenteufel & Quintard, 2022). Minimising parents' regret is also essential for healthy bereavement, as unresolved regret can complicate the grieving process. Parents who had minimal involvement in their infant's bedside care expressed feelings of regret and guilt after their loss ( Sim *et al*., 2020). Clarke & Connolly (2022) found that parents appreciated memory-making activities, such as creating personalised memory boxes, hand and footprint jewellery, and moulds, which help document their child's life and maintain a connection after their death, significantly aiding the grieving process. The National Policy on Palliative Care for Children with Life-Limiting Conditions in Ireland ( DoHC, 2009) and studies such as Clarke & Connolly (2022) highlight the significance of interventions like memory-making interventions, which allow families to create lasting memories and maintain a continuing bond with their child.

This review focuses on children and young people aged 0–19 years with LLLT conditions, recognizing that care and support needs vary significantly across different age ranges, developmental stages, and conditions. Younger children may require more family involvement in interventions, such as creating scrapbooks and memory boxes, while adolescents and young adults might engage more independently in activities like hobbies and school. Understanding these differences ensures that interventions are tailored to support infants, children, young people and their families effectively ( Coombes *et al*., 2022).

Memory-making interventions are increasingly recognised within paediatric palliative and bereavement care contexts to help children and families make meaning out of their experiences ( Akard *et al.*, 2021a; Akard *et al.*, 2021b; Sisk *et al.*, 2012). Memory-making, also often referred to as creating a legacy ( Akard *et al.*, 2021c), is a broad term describing specific processes, such as sense-making and benefit finding, that contribute to adapting to stressful life experiences and is a core element in coming to terms with grief ( Kobler *et al.*, 2007; Park, 2013). Memory-making interventions can include tangible activities such as memory boxes, handprint art, or digital storytelling and recording special messages. These interventions serve not only as therapeutic tools to support emotional expression and processing but also as valuable artifacts that families can hold onto, serving as a source of comfort and connection to their loved one ( Boles & Jones, 2021).

Four recent review papers have shed light on the diverse approaches to memory-making in healthcare ( Boles & Jones, 2021; Keller *et al.*, 2024; MacEachen *et al.*, 2023; Xu *et al.*, 2024). Although these reviews cover a broad spectrum, none specifically focused on memory-making interventions for children with LLLT conditions and their families within the realms of palliative or bereavement care. Boles and Jones (2021) highlighted the significant benefits of legacy interventions, for both adult and paediatric populations, showing significant improvements in well-being and a reduction in depression symptoms among adults. This review demonstrates the utility and benefits of legacy interventions but highlights the scarcity of paediatric focused research and the need for standardized practices in legacy interventions. Keller *et al.* (2024) took a deep dive into what "legacy" means in the context of paediatric healthcare. They identified that legacy creation in children's healthcare is a collaborative process that leaves something lasting to remember the child by, capturing the unique essence or spirit of the child. They identified that legacy-oriented interventions are provided in most children's hospitals in the United States yet highlighted the absence of a widespread consensus on the foundation or scope of these interventions. This gap emphasises the critical need for evidence-based guidelines and practices in creating and implementing legacy for and with children, suggesting an opportunity to enhance the quality of care for children and their families globally.

In a qualitative thematic synthesis, MacEachen *et al.* (2023) explored families' experiences of memory-making in both adult and paediatric critical care settings, aiming to understand the impact on bereaved families. The synthesis identified four main themes describing families' experiences: connection, compassion, engagement and creation, and continuation. MacEachen’s review illuminates how memory-making facilitates a connection between families and healthcare staff, providing a structure and purpose during the bereavement process, and fostering a continuing bond with the deceased. The review also highlights the lack of research focusing on paediatric critical care settings, marking a vital area for future exploration. Finally, Xu *et al.* (2024) conducted a qualitative synthesis examining bereaved parents' perceptions of memory-making. Their findings reveal parents' recognition of memory-making's significance in connecting with their children and navigating grief. Yet, they identified barriers such as a lack of understanding and preparation, along with limited support, which sometimes led parents to miss out on these meaningful opportunities. The review calls for a deeper investigation into how memory-making practices and intervention can be better tailored to meet the diverse needs of families, particularly considering cultural sensitivity and the evolving nature of grief over time. While these previous reviews have revealed important information about memory-making and memory-making interventions in various contexts and with different populations, they also highlight significant gaps in our understanding, particularly concerning paediatric populations, and the need to consider challenges faced by bereaved parents. To date, to our knowledge, no systematic review has been undertaken to specifically examine memory-making interventions for children with LLLT conditions and their family receiving palliative and/or bereavement care.

Therefore, the aim of this review is to identify, appraise, and synthesise the current evidence on memory-making interventions for children with LLLT conditions and their family members who are receiving palliative or bereavement care. Our objectives include synthesizing evidence on the (1) range of memory-making interventions used for children with LLLT conditions and their families receiving palliative or bereavement care, (2) effectiveness of memory-making interventions by synthesizing outcomes reported in the literature and mapping the measures used to assess psychosocial outcomes like resilience, emotional coping, family communication, and any other relevant outcomes, (3) experiences of families who participate in memory-making interventions, focusing on their perceptions, the perceived impact on their emotional and psychosocial well-being, and the contribution to family cohesion, and (4) barriers and enablers affecting the successful implementation of memory-making interventions.

## Protocol

### Methods

This protocol was developed in accordance with the guidelines for the Preferred Reporting Items for Systematic Reviews and Meta-Analysis Protocols Checklist (PRISMA-P) ( Moher *et al.*, 2015). This review protocol has been registered prospectively on the PROSPERO database for systematic reviews (registration number CRD42024521388; 18/03/2024). This systematic review will be reported in accordance with the Preferred Reporting Items for Systematic Reviews and Meta-Analysis (PRISMA) statement ( Page *et al.*, 2021).

### Eligibility criteria

The eligibility criteria for inclusion in this review will be outlined using the Population, Intervention, Comparison, Outcome (PICO) framework.

### Population

This review targets children and young people aged 0–19 years with LLLT conditions and their families. A life-limiting condition is defined as an illness or disorder with no reasonable expectation of cure, leading to premature death and requiring comprehensive care that addresses the child’s physical, psychological, social, and spiritual well-being. A life-threatening condition, conversely, is characterized by a significant risk of death but may be amenable to curative treatment or prolonged management. This review extends to family members involved in or benefiting from memory-making interventions in the context of palliative or bereavement care. Family members include, but are not limited to, parents, siblings, and other relatives, as identified by the studies included in this review.

### Interventions

Memory-making interventions are identified as therapeutic activities designed to aid children with LLLT conditions, and their families, in crafting lasting memories. These interventions encompass a broad range of activities, including digital storytelling, the creation of physical mementos, and participatory art projects. The interventions focus on enhancing emotional well-being, offering comfort, and bolstering coping and resilience in the face of adversity. This review considers interventions that involve the child alone, child-parent dyads, child-other family member dyads, child-parent-other family member triads, the entire family unit, and interventions for bereaved family members. The scope includes interventions directed by, with, or about children and their families, aiming to support them during palliative care or the bereavement process.

### Comparators/control

This review will compare memory-making interventions against usual care practices or other active interventions as defined by included studies. In qualitative or exploratory studies without direct comparators, no specific control will be applied.

### Main outcome(s)

The primary focus of this systematic review is on psychosocial outcomes, which are broadly conceptualized to cover various aspects of psychosocial outcomes for children with LLLT conditions and their families. These outcomes include, but are not limited to:

•   Quality of life,

•   Mental health indicators such as stress, anxiety, and depression,

•   Aspects of communication within the family and strength of family communication.

### Additional outcome(s)

Beyond the primary focus, this review also seeks to explore the wider impacts of memory-making interventions on families and children with LLLT conditions. These additional outcomes include, but are not limited to:

•   Coping and resilience mechanisms developed because of the interventions,

•   Satisfaction with and perceived value of the intervention by participants,

•   The role of these interventions in the creation and preservation of memories.

### Study type

This review will include studies that provide qualitative, quantitative, or mixed-method data, including but not limited to randomized controlled trials, cohort studies, case-control studies, cross-sectional studies, qualitative studies, and mixed-methods studies. This approach allows for a broad synthesis of evidence across diverse research designs, offering a comprehensive understanding of memory-making interventions within paediatric palliative and bereavement care.

## Information sources and search strategy

To ensure a comprehensive literature review, we will conduct searches across multiple databases for peer-reviewed publications from January 1, 1985, to February 27, 2024. These databases include PubMed, EMBASE, CINAHL (EBSCO), PsycINFO (EBSCO), Web of Science, Cochrane Library, and Scopus. Additionally, we will examine the reference lists of related reviews to identify further studies that meet the review eligibility criteria.

The search strategy of this review has been systematically developed according to the PICO framework, focusing on the Population (P) and Intervention (I) components. This involved the utilization of the MeSH database in PubMed and Emtree via Embase and was informed by previous systematic reviews. A sample search strategy developed with a librarian is presented in Table 1.

**Table 1.** Sample search strategy.

Search ID	Search Terms
S1	legacy OR "legacy making" OR "legacy-making" OR "memory making" OR "memory-making" OR "legacy building*" OR "legacy activit*" OR "dignity therap*" OR "legacy service*" OR "life review"
S2	child* OR teen* OR youth* OR adolescent* OR "under 19" OR "under 18" OR infant* OR newborn* OR "new born*" OR "new-born*"
S3	pediatric* OR paediatric* OR parent* OR sibling* OR famil* OR "family members"
S4	S2 OR S3
S5	intervention* OR therap* OR treatment* OR "activit*"
S6	bereavement OR bereave* OR grief OR mourning OR "grief support" OR "mourning support
S7	S5 OR S6
S8	S1 AND S4 AND S7

### Screening and selection of studies

Search outputs will be imported into Covidence where a two-part screening process will be undertaken once duplicates are removed. Part one screening will involve two reviewers (RS, VL) independently screening titles and abstracts against the review eligibility criteria. Any discrepancies between reviewers will be resolved through consensus or discussion with a third reviewer (EC). For part two screening, two reviewers (RS, VL) will independently assess full texts against the eligibility criteria before a final decision regarding inclusion or exclusion is confirmed. Any discrepancies will be resolved through consensus or discussion with a third reviewer (EC) acting as arbitrator as required. We will record reasons for excluding studies at the full-text stage. An adapted PRISMA flow chart will be used to report the screening and selection process at each stage of the review.

### Data extraction

Data extraction will be conducted by one reviewer (RS) using designed data extraction forms tailored to each study type (e.g., quantitative, qualitative, mixed methods), which will be refined based on pilot data extraction. Across the included studies, where relevant, data will be extracted on authors, title, year of publication, country, aim, study design, participant demographics, interventions and comparators, outcome measures, and key findings. Where we encounter missing, unclear, or incomplete data, we will contact the study authors to obtain additional information. Extracted data will be cross-checked by a second reviewer (VL) for accuracy and completeness. Any discrepancies will be resolved through consensus, or, if necessary, through consultation with a third reviewer (GK).

### Quality appraisal

Utilizing the Mixed Methods Appraisal Tool (MMAT), two independent reviewers (RS, YC) will appraise the methodological quality of the included studies. This appraisal will focus on the robustness of the study design, data collection, and analysis. The MMAT's versatility allows for a detailed appraisal across different study types (i.e., qualitative, quantitative, mixed methods etc.), ensuring a thorough examination of bias and methodological integrity ( Hong *et al.*, 2018). In instances of divergent assessments, consensus will be sought through discussion or consultation with a third reviewer (VL).

### Data analysis and synthesis

Joanna Briggs Institute (JBI) guidelines for conducting mixed methods systematic reviews will be used to inform the data analysis and synthesis process. By adhering to the JBI Manual for Evidence Synthesis, we plan to integrate quantitative and qualitative findings effectively ( Peters *et al.*, 2020). Through narrative synthesis, we will assess the effectiveness, implementation, and quality of memory-making interventions in palliative care, identifying common patterns, themes, and points of divergence across studies. Where feasible, quantitative data may be subjected to meta-analysis to examine intervention outcomes, provided that the data demonstrate sufficient homogeneity. Qualitative synthesis, on the other hand, will focus on identifying barriers, facilitators, and the contextual factors influencing the efficacy and implementation of interventions. This process will be informed by the Template for Intervention Description and Replication (TIDieR) checklist and JBI's convergence framework for mixed methods studies, ensuring clarity in intervention description and a nuanced understanding of the combined evidence ( Hoffmann *et al.*, 2014). This review's goal is to provide a comprehensive synthesis that reveals the complex dimensions and impacts of interventions, thereby informing practice and guiding future research on targeted intervention strategies.

## Conclusion

This systematic review will culminate in a comprehensive synthesis of evidence on memory-making interventions in paediatric palliative and bereavement care, highlighting psychosocial and other impacts, implementation factors, and evidence quality. By identifying best practices and gaps in knowledge, this evidence review may inform future research and intervention design, or adaptation, and contribute to the enhancement of future healthcare for children and young people with life-limiting and life-threatening conditions and their families, as well as those in bereavement.

## Data Availability

No underlying data are associated with this article. A set of extended data associated with this article includes the Protocol registered with the International Prospective Register of Systematic Reviews (PROSPERO). The registration ID is CRD42024521388, which can be viewed at: https://www.crd.york.ac.uk/prospero/display_record.php?ID=CRD42024521388 PRISMA-P Checklist associated with this article is available on OSF, under the project title “Memory-making interventions for children and their families receiving pediatric palliative or bereavement care”, https://doi.org/10.17605/OSF.IO/79JV5 ( Safarifard *et al.*, 2024) Data are available under the terms of the Creative Commons Zero "No rights reserved" data waiver (CC0 1.0 Public domain dedication).
