# Supplementary material for: Memory-making interventions for children and their families receiving pediatric palliative or bereavement care: A systematic review protocol
Source: HRB Open Res. 2024 Jul 17;7:30. Originally published 2024 May 20. [Version 2] doi: 10.12688/hrbopenres.13891.2 (PMC11282391; doi:10.12688/hrbopenres.13891.2)
Supplement: Peer review history

# Reviewer report: Reviewer response for version 2

**DOI:** 10.21956/hrbopenres.15282.r41385

**Published:** 2024-07-26

**Authors:** Myra Glajchen

**Affiliations:** 1 Albert Einstein College of Medicine, Bronx, New York, USA

The revised version of this manuscript is responsive to the issues raised by both reviewers. The literature review is more comprehensive with important definitions of palliative care, legacy building, bereavement, age range for pediatrics, and interventions for both pediatric patients and their caregivers. With these revisions, I hereby give my approval for indexing.

Is the study design appropriate for the research question?

Yes

Is the rationale for, and objectives of, the study clearly described?

Yes

Are sufficient details of the methods provided to allow replication by others?

No

Are the datasets clearly presented in a useable and accessible format?

Partly

Reviewer Expertise:

Psychosocial oncology, palliative care, oncology care, social work, interprofessional training, caregiver burden./

I confirm that I have read this submission and believe that I have an appropriate level of expertise to confirm that it is of an acceptable scientific standard.

# Reviewer report: Reviewer response for version 2

**DOI:** 10.21956/hrbopenres.15282.r41384

**Published:** 2024-07-18

**Authors:** Alison Rodriguez

**Affiliations:** 1 University of Leeds, Leeds, England, UK

Dear Authors, thank you for considering the suggested amendments. I think the article reads well and is a valuable contribution to the evidence base.

Is the study design appropriate for the research question?

Yes

Is the rationale for, and objectives of, the study clearly described?

Yes

Are sufficient details of the methods provided to allow replication by others?

Yes

Are the datasets clearly presented in a useable and accessible format?

Yes

Reviewer Expertise:

Health psychology - Children and young people with life limiting and life threatening conditions, lived experience, life review and meaning making, phenomenology, long term conditions, and self management.

I confirm that I have read this submission and believe that I have an appropriate level of expertise to confirm that it is of an acceptable scientific standard.

# Reviewer report: Reviewer response for version 1

**DOI:** 10.21956/hrbopenres.15233.r40149

**Published:** 2024-06-18

**Authors:** Alison Rodriguez

**Affiliations:** 1 University of Leeds, Leeds, England, UK

Thank you for the invitation to review this article. The manuscript details a well-structured review protocol that aims to identify, appraise, and synthesise the evidence base that reports on memory making activities for children and young people (CYP) (0-19yrs) with life-limiting and life-threatening conditions (LLLTC) and their families within both palliative and bereavement pediatric care contexts. These activities are undertaken in practice to provide some level of emotional and psychosocial support to CYP and families. A review of this nature is welcomed. The authors draw on relevant review guidance and propose methods to safeguard the reliability/trustworthiness of the review procedures and findings. There are just a few points that the authors may want to further consider:

1. The abstract of the protocol summarises the protocol well but there could be further acknowledgement of bereavement care in the conclusion section given the focus is on memory making activities in palliative and bereavement care for CYP and their families.
2. In the introduction section of the protocol, it is stated that there are difficulties with accurately estimating prevalence rates for the population under study. This is true but it would be useful for the readership to know if there are any current and projected estimates proposed? (To further consider how impactful these types of interventions can/could be).
3. It is unclear what age range this work is focused upon until we get to the methods section of the protocol. Given the age range is 0-19 there could be some earlier acknowledgement of this in the introduction and some consideration of how care and support may be different for CYP of different age ranges (within this 0-19 range), and how support for families and their involvement in care maybe different for different age ranges, developmental stage/ability, and condition type. The authors are considering a patient group that includes many conditions with a multitude of impacts in terms of life expectation, related disease trajectories, emotional and psycho-social needs, and that may engage different models of care. Also, where some CYP will be able to engage in interventions independently, others will not. The review undoubtedly will unveil issues relevant to these points, but I think an acknowledgement of the variance in support need and levels of CYP participation in these types of activities may be worthwhile.
4. Use of the terms ‘children and young people’ is generally preferred over just ‘children’ given the proposed age range includes adolescents and young adults.
5. Within the method section there is narrative around how appraisal of any included study will acknowledge standard care practices. It will be interesting to see if studies have done this in exploring these types of interventions within paediatric care because there is no standard of care with respect to emotional and psycho-social support for these CYP.
6. If the review will be limited to paediatric settings, there may be potential to exclude studies reporting on memory making interventions that could be accessed by CYP transitioning to adult care services and intervention studies that have included people above 18 years of age in their inclusion criteria. If the authors remain happy to exclude these studies, then perhaps a stronger case to only include studies that have explored memory making within paediatric care settings could be afforded. It would be useful for the authors to at least note how models of care can be different and this may mean some CYP and families access memory making interventions outside of paediatric services.
7. In terms of considering outcome measures it is good that the authors have considered to ‘include but not be limited to’ criteria as it is expected that a wide array of outcomes could be reported (and not necessarily measured).

Is the study design appropriate for the research question?

Yes

Is the rationale for, and objectives of, the study clearly described?

Yes

Are sufficient details of the methods provided to allow replication by others?

Yes

Are the datasets clearly presented in a useable and accessible format?

Yes

Reviewer Expertise:

Health psychology - Children and young people with life limiting and life threatening conditions, lived experience, life review and meaning making, phenomenology, long term conditions, and self management.

I confirm that I have read this submission and believe that I have an appropriate level of expertise to confirm that it is of an acceptable scientific standard.

# Response

**Published:** 2024-07-03

**Authors:** Razieh Safarifard

Dear Reviewer,

 Thank you for your thoughtful and constructive feedback on our manuscript. We appreciate your positive comments and have carefully considered your suggestions. Below, we address each of your points and outline the revisions made in the second version of our protocol:

 1. Bereavement Care in the Conclusion Section of the Abstract: The conclusion has been revised to explicitly include bereavement care, enhancing the clarity and scope of the abstract.

 2. Prevalence Rates in the Introduction: We added current and projected estimates of children with life-limiting conditions to the introduction, highlighting the growing demand for palliative and bereavement care.

 3. Acknowledgement of Age Range and Support Needs: The age range (0-19 years) has been clarified, and the background section of the protocol has been expanded to detail the diverse support needs across different ages and developmental stages.

 4. Terminology - Children and Young People (CYP): The manuscript was reviewed to ensure the term "children and young people" is used consistently and appropriately throughout the text.

 5. Standard Care Practices in Methods Section: The term "standard care" has been reworded to "usual care" to better capture studies that report any standard regarding the intervention, clarifying our evaluation criteria.

 6. Focus on Paediatric Settings: Thank you for your comment and suggestion. We are not just focused on paediatric palliative care but are also interested in other relevant settings to provide a comprehensive review.

 7. Outcome Measures: We are grateful for your acknowledgment of the inclusiveness of our approach to outcome measures. We concur that a wide array of outcomes, both reported and measurable, is essential for a comprehensive systematic review.

 We value your insights, which have significantly enhanced our manuscript. We look forward to continuing this productive dialogue.

 Sincerely,

# Reviewer report: Reviewer response for version 1

**DOI:** 10.21956/hrbopenres.15233.r40150

**Published:** 2024-06-06

**Authors:** Myra Glajchen

**Affiliations:** 1 Albert Einstein College of Medicine, Bronx, New York, USA

Memory-making is an important intervention in oncology, palliative care and hospice. According to this review, such interventions confer benefits for children with life-limiting conditions. This timely systematic review aims to fill an important gap in the literature given that this area has been under-studied. Unfortunately, the review is not scientifically sound, literature is lacking in lacking critical areas including palliative care, caregiver distress, minimizing caregiver regret as part of healthy bereavement, and understanding meaning-making from a developmental perspective. In addition, it is impossible to assess the quality of the data given that the authors do not share their findings. The utility of this paper lies in its list of references and description of its methodology.

Is the study design appropriate for the research question?

Yes

Is the rationale for, and objectives of, the study clearly described?

Yes

Are sufficient details of the methods provided to allow replication by others?

No

Are the datasets clearly presented in a useable and accessible format?

Partly

Reviewer Expertise:

Psychosocial oncology, palliative care, oncology care, social work, interprofessional training, caregiver burden./

I confirm that I have read this submission and believe that I have an appropriate level of expertise to state that I do not consider it to be of an acceptable scientific standard, for reasons outlined above.

# Response

**Published:** 2024-07-03

**Authors:** Razieh Safarifard

Dear Reviewer,

 Thank you for your thoughtful review of our systematic review protocol paper. We appreciate your feedback and have made several revisions to address your concerns and enhance our work.

 We have expanded the literature to define palliative care more clearly, emphasizing its role in improving the quality of life for both the child/young person and their families. We acknowledge the emotional burden on caregivers in paediatric palliative care and the importance of minimizing caregiver regret for healthy bereavement. Additionally, we have highlighted that memory-making interventions help families create meaningful memories, reducing feelings of regret and supporting a healthier grieving process. We refined our background section to emphasize the critical areas you mentioned. Our protocol now reflects these concerns more clearly.

 Regarding your comment on the impossibility of assessing the quality of the data due to the lack of shared findings, we acknowledge the challenge. As this is a protocol for a systematic review, we appreciate the difficulty in assessing data quality at this stage. We will report more explicitly on this aspect in the findings once the systematic review is completed.

 We appreciate your valuable insights, which have significantly helped us improve our manuscript.

 Sincerely,
